# A Novel Antioxidant Protects Against Contrast Medium-Induced Acute Kidney Injury in Rats

**DOI:** 10.3389/fphar.2020.599577

**Published:** 2020-11-27

**Authors:** Shuo Huang, Yanyan Tang, Tianjun Liu, Ning Zhang, Xueyan Yang, Dingwei Yang, Ge Hong

**Affiliations:** ^1^ Clinical College of Orthopedics, Tianjin Medical University, Tianjin, China; ^2^ Tianjin Key Laboratory of Biomedical Materials, Institute of Biomedical Engineering, Chinese Academy of Medical Science and Peking Union Medical College, Tianjin, China; ^3^ School of Chemical Engineering, Anhui University of Science and Technology, Huainan, China; ^4^ Department of Nephrology, Tianjin Hospital, Tianjin, China

**Keywords:** contrast-induced acute kidney injury, apoptosis, oxidative stress, mitochondrial dysfunction, antioxidant

## Abstract

Many studies proposed that oxidative stress and apoptosis are key mechanisms in the pathogenesis of contrast-induced acute kidney injury (CI-AKI). Xylose-pyrogallol conjugate (XP) is an original effective antioxidant that showed decent antioxidant and anti-apoptosis effect before. Thus the therapeutic effect and mechanism of XP in preventing CI-AKI in the short and long term were investigated in this research. Renal function and histological grade were evaluated to determine the severity of renal injury. Kidney samples were then collected for the measurement of oxidative stress markers and the detection of apoptosis. Transmission electron microscopy (TEM) and western blot of mitochondrial protein were utilized for the analysis of the mitochondrial conditions. The results demonstrated that the CI-AKI rats caused a significant decrease in renal function accompanied by a remarkable increase in Malondialdehyde (MDA), bax, caspase-3, cytochrome c (Cyt C) level, TdT-mediated dUTP nick end labeling (TUNEL) positive apoptotic cells, and damaged mitochondria, while a decline in antioxidase activities and mitochondrial superoxide dismutase 2 (SOD2) expression compared with the control rats. However, when XP (50 or 100 or 200 mg/kg/day) was given orally for consecutive 7 days before CI-AKI modeling, XP (200 mg/kg) showed a better capability to restore renal dysfunction, histopathological appearance, the level of apoptosis, mitochondrial damage, oxidative stress, and fibrosis generation without interference in computed tomographic imaging. Our study indicated that antioxidant XP played a nephroprotective role probably via antiapoptotic and antioxidant mechanisms. Besides, XP may regulate the mitochondria pathway via decreasing the ratio of bax/bcl-2, inhibiting caspase-3 expression, cytochrome c release, and superoxide dismutase 2 activity. Overall, XP as a high-efficient antioxidant may have the potentials to prevent CI-AKI.

## Introduction

Contrast-induced acute kidney injury (CI-AKI) is caused by iodinated contrast media for diagnostic imaging (Azzalini et al., 2016) and it is the third most common reason of hospital-obtained AKI, occurring in more than 30% of patients receiving iodinated contrast media injection, and it is related to a high risk of mortality caused by renal dysfunction (Fahling et al., 2017). Though most patients with normal renal function do not experience renal complications after receiving contrast, those with redued volume or chronic kidney disease (CKD) have a higher risk of CI-AKI (Khwaja, 2012; Weisbord et al., 2018). CI-AKI occurs within 24–72 h following the radiocontrast agents administration and it is correlated to adverse outcomes including acute renal failure requiring dialysis and worsening of CKD (Khwaja, 2012). Unfortunately, the exact pathogenesis of CI-AKI remains unclear, which leads to a lack of effective prevention and treatment methods for CI-AKI. However, the major mechanism of CI-AKI may be associate with direct cytotoxic induced apoptosis effects on renal tubular, mitochondrial dysfunction, and oxidative stress (Khwaja, 2012). Numerous preclinical researches showed that protective agents with antioxidant activity could prevent the occurrence of CI-AKI by reducing tubular epithelial cells apoptosis, mitochondrial reactive oxygen species (ROS) production, DNA oxidative damage in kidney tissues (Dennis and Witting, 2017). Thus, a probable treatment strategy may include the use of drugs targeting the regulators of apoptosis, mitochondria and renal oxidative stress.

As our team previously reported, aiming to find novel high-efficiency antioxidants, 32 epigallocatechin gallate (EGCG) analogs were synthesized and characterized (Liu, 2012). Among them, Xylose-pyrogallol conjugate (XP) displayed strong free radical scavenging capacity and it could protect EVC304 cells from hydrogen peroxide-induced oxidative damage *in vitro* (Liu, 2012). XP is a polyphenol based compound with excellent water solubility and its chemical structure is the most similar to EGCG. EGCG, as one of the extracts in green tea, has been known for possessing antioxidant, anti-apoptosis, and anti-fibrosis characteristic in the diseases of cardiovascular system, lung (Takahashi et al., 2009), liver (Niu et al., 2013; Kochi et al., 2014) and urinary system (Niu et al., 2013; Kochi et al., 2014). Moreover, EGCG have been verified in either acute or chronic kidney diseases, including cisplatin-induced nephrotoxicity (Sahin et al., 2010; Pan et al., 2015), ischemia-reperfusion AKI (Kaviarasan et al., 2007), and diabetic nephropathy (Kaviarasan et al., 2007). Besides, more and more researches have revealed that mitochondrial damage has been thought highly in apoptosis (Kaviarasan et al., 2007) and tubular cells have plentiful mitochondria. Therefore, we hypothesized that XP may exert a protective effect as a new antioxidant on the CI-AKI rat model through the regulation of mitochondrial function, oxidative stress, and apoptosis.

## Materials and Methods

### Reagents and Antibodies

The iodinated radiographic contrast agent used in this study, iohexol (300 mg/ml iodine) was purchased from Beilu Pharmaceutical Co. Ltd. (Beijing, China). Indomethacin was obtained from Sigma-Aldrich (St. Louis, MO, Unites States). Nω-Nitro-L-arginine methyl ester hydrochloride was obtained from Aladdin Biochemical Technology Co., Ltd. (Shanghai, China). The kits of malondialdehyde (MDA), superoxide dismutase (SOD), glutathione peroxidase (GSH-Px) and catalase (CAT) were obtained from Beyotime Biotechnology (Shanghai, China). The TdT-mediated dUTP nick end labeling (TUNEL) detecting kit was purchased from Roche Molecular Biochemicals (Mannheim, Gemany). Primary antibodies against bax, bcl-2, caspase-3, cytochrome c oxidase IV (COX IV), superoxide dismutase 2 (SOD2) and cytochrome c (Cyt C) were obtained from Abclonal Biological Technology Co., Ltd. (Wuhan, China). Alexa Fluor 488-conjugated secondary antibody was purchased from R&D Systems (Minneapolis, MN, Unites States). Horseradish peroxidase (HRP)-conjugated anti-rabbit antibody and diaminobenzidine tetrahydrochloride (DAB) were purchased form Zsbio Biotechnology Co., Ltd. (Beijing, China). Western blotting enhanced chemiluminescence (ECL) was provided by Abbkine scientific Co., Ltd. (California, Unites States). Diamidino-2- phenylindole (DAPI) and BCA^™^ protein assay kit were bought from Solarbio Life sciences (Beijing, China). Dimethyl sulfoxide (DMSO) and other chemicals were bought from Tianjin Jiangtian Chemical Technology Co., Ltd. (Tianjin, China).

### Preparation and Circular Dichroism Spectral Analysis of Xylose-Pyrogallol

The chemical name of XP was 1-Deoxy-1,1-bis(2,3,4-trihydroxyphenyl)-D-xylitol. The synthetic process of XP was realized according to the patent specifications (No. ZL201210519507.9) by Prof. Liu from Institute of Biomedical Engineering, CAMS (Liu, 2012). Firstly, xylose (1 equiv) and pyrogallol (4 equiv) were added to a 500 ml flask and heated at 120°C in a nitrogen atmosphere. The molten mixture was stirred for 24 h at a fixed temperature. The final product XP (the purity ≥ 95%) was obtained by crystallizing the resulting light grey solid in the mixed solution of methanol and ethyl acetate. In addition, XP solution was prepared in water to get a 0.5 mM concentration and the Circular Dichroism (CD) spectra were recorded on a JASCO J-815 automatic spectrophotometer (Jasco Corporation, Tokyo, Japan) in cells of 1.0 mm path length at 25°C. The spectra were measured from 200 to 350 nm, with a scan rate of 200 nm/min. All the chemicals and reagents used in the preparation of XP were obtained from Sigma-Aldrich (St. Louis, MO, Unites States). They were of analytical grade and used without any purification.

### Experimental Animals

Male Sprague–Dawley rats (180 ± 5 g) were purchased from HuaFuKang Bioscience Co., Ltd. (Beijing, China). All animal experiments procedures were experimented according to the National Institutes of Health Guide for Care and Use of Laboratory Animals, and the protocol was approved by the Laboratory Animal Management Committee/Laboratory Animal Welfare Ethics Committee, Institute of Radiation Medicine, Chinese Academy of Medical Sciences. The rats were housed in a ventilated and standardized animal room with controlled conditions of light (12 h light/12 h dark cycle), temperature (23 ± 2°C) and humidity (55 ± 2%). They were acclimated to this environment for 5 days prior to the start of the experiments and had free access to plenty of standard chow and tap water.

### Modeling and Grouping

Rats were subcutaneously injected with indomethacin (IN) dissolved in DMSO (10 mg/kg) and L-NAME dissolved in 0.9% saline (10 mg/kg) after overnight (12 h) water deprivation (Billings et al., 2008). That caused systemic especially renal vasoconstriction and modeled the early stage of renal impairment. Then rats were administered iohexol (3 g iodine/kg) via the caudal vein 15 min later. Subsequent 24 h, 1 ml venous blood was collected from each anesthetized rat, and serum was centrifugated for use in creatinine assessments. The result manifested >50% increase in serum creatinine from baseline (or reached a 1.5 fold baseline level), therefore, it was considered a successful CI-AKI model (Thomsen and Morcos, 2003). Moreover, this model has been previously validated in mice (Lee et al., 2006).

The experiment was separated into two parts. The protective effect of the XP pretreatment in CI-AKI and fibrosis intervening function of XP within 14 days of contrast medium induced kidney injury were evaluated in a two-stage experiment. Firstly, a total of 60 Sprague–Dawley rats were weighed and then randomly divided into six groups: (1) saline-treated control group (Ctrl, *n* = 10), (2) XP (200 mg/kg/day, dissolved in 0.9% saline) alone treated group (XP, *n* = 10), (3) contrast medium (CM) treated group (CI-AKI, *n* = 10), (4) low dose XP (50 mg/kg/day) plus CM treated group (LXP, *n* = 10), (5) medium dose XP (100 mg/kg/day) plus CM treated group (MXP, *n* = 10), and (6) high dose XP (200 mg/kg/day) plus CM treated group (HXP, *n* = 10). The rats were administrated with or without XP orally for seven consecutive days before applying CM. Furthermore, CM treated rats were processed in accordance with the protocol described above 24 h after contrast administration, six animals of each group were chosen randomly and anesthetized with 10% chloral hydrate (0.3 ml/100 g) and were collected venous blood for further serological assays, preceded by euthanasia, body mass measurement and kidneys removal after perfusion. Next, the kidneys were weighted and bisected in the coronal plane; the right kidney was divided for western blot and oxidative stress analyses, and the left kidney was fixed in 4% paraformaldehyde (PFA) and prepared for routine histological examination.

The left four rats in each group, total 24 rats were then given free access to food and water without any intervention and killed 14 days later form Iohexol injection for kidney removal to observe the fibrosis degree via Masson staining. In order to distinguish the groups from the former, their name were changed as following: (1) Con-Vehicle, *n* = 4, (2) Con-XP, *n* = 4, (3) CM-Vehicle, *n* = 4, (4) CM-LXP, *n* = 4, (5) CM-MXP, *n* = 4, (6) CM-HXP, *n* = 4.

### Biochemical Evaluation of Blood and Urine Samples

Blood samples were collected through orbital sinus 24 h after modeling and allowed to clot spontaneously at room temperature. Serum was centrifugated at 3,000 × *g* for 10 min at 4°C. Rats were put in metabolic cages after iohexol injection and urine samples were collected 24 h later. Serum and urine samples were submitted for measurements on the same day of collection. Blood urea nitrogen (BUN), serum creatinine (Scr), cystatin C (Cys C), and urinary N-acetyl-β-D-glucosidase (uNAG) were analyzed using automatic biochemistry analyzer Siemens ADVIA^®^2400 (Munich, Germany).

### Morphology and Pathology Evaluations of Kidney Tissues

Four to six micrometer whole kidney sections were deparaffinized in xylene, rehydrated in a graded series of ethyl alcohol and then stained with hematoxylin and eosin (HE) and Periodic Acid-Schiff (PAS) for general morphological evaluation by the Biossci Biotechnology Co., Ltd. (Wuhan, China), and images were scanned using a NanoZoomer S360 (Hamamatsu, Japan). Tubular injury scores were assigned to HE-stained and PAS-stained kidney sections by two experienced kidney pathologists from outside who were blinded to the identity of the samples through selecting randomly 10 consecutive (×400) fields of cortex and outer stripe of medulla from each section. We took published criteria as a description for each parameter including vacuolization, luminal cell casts, and atrophic changes (Hur et al., 2013). The relative severity of each lesion was graded from 0 to 4 and total pathology score was calculated. Tubular injury/degeneration was graded as following: no damage (−or 0), mild (+− or 1, unicellular, patchy isolated damage), moderate (+or 2, damage <25%), severe (++ or 3, damage between 25 and 50%), and very severe (+++ or 4, damage >50%) (Hur et al., 2013). The PAS-stained section had additional tubulointerstitial inflammation score that defined as the presence of inflammatory infiltration in interstitial areas, and the scoring criterion was identical to the above. Fourteen days later-tissue samples (4–6 μm) were stained with Masson’s trichrome to evaluate the level of fibrosis. The microscope image was digitized using Image J software and the digital image of the signal was quantified as collagen volume fraction (CVF). The CVF was calculated as the ratio of the sum of the total area of interstitial fibrosis to that of the total connective tissue area plus the renal glomerular and tubular in the entire visual field of the section (Lin et al., 2019a).

### Indirect Immunofluorescence Staining

Tissues were collected after perfusion, then fixed in 4% PFA, placed in 18% sucrose in phosphate buffer saline (PBS) at 4°C overnight consecutively, and embedded in optimal cutting temperature (OCT) compound. Five µm micrometer frozen tissue sections were air-dried at room temperature (RT) and sequentially washed three times for 15 min each with 0.1 M of cold PBS solution containing 2% Triton X-100, followed by incubation for 2 h at ambient temperature with a blocking solution containing 10% normal goat serum diluted with 0.1% bovine serum albumin in PBS. Then tissues sections were incubated with anti-bax, anti-caspase-3 and anti-bcl-2 primary antibodies (1:100 diluted with 0.1% bovine serum albumin) overnight at 4°C, and with secondary antibody (1:100 diluted with 50% glycerol) sheltered from light for 1 h at RT, and nuclei were stained by Diamidino-2- phenylindole. Images were acquired using Carl Zeiss LSM710 confocal laser-scanning microscope (Jena, Germany) with constant acquisition parameters.

### TdT-Mediated dUTP Nick End Labeling Assay

In order to assess the apoptotic cells, tissue sections were stained by fluorometric TUNEL system in accordance with the manufacturer’s instructions and scanned under NanoZoomer S360. 10 random fields of the cortex region of kidney were imaged under high magnification (× 400) using CaseViewer software and quantified by Image J. Divide the number of TUNEL positive cells by the total number of cells to obtain the percentage of TUNEL positive cells.

### Analysis of the Malondialdehyde and Antioxidant Markers

The lipid peroxidation (LPO) statues were measured by the thiobarbituric acid reactive substances (TBARS) method and were shown as MDA concentrations. Serum samples were assayed by the MDA Assay Kit using a multifunctional microplate spectrophotometer Thermo 3,001 (Thermo Fisher Scientific, Waltham, MA, United States). The MDA level was expressed as μmol/L. The activity of (SOD, GSH-Px, and CAT in renal tissues were measured using the total superoxide dismutase assay kit with WST-8, total glutathione peroxidase, and catalase assay kit respectively. Kidney tissues were homogenized on ice with detection buffer according to the manufacturer’s instructions and using a Thermo 3001 microplate spectrophotometer at corresponding absorption wavelength to obtain optical density values. Finally, the results were converted to U/mg-protein, mU/mg-protein, U/mg-protein respectively.

### Immunohistochemistry

The paraffin-embedded kidney sections were deparaffinized and rehydrated as above described, then boiled in citrate antigen retrieval solution (PH = 9) for 3 min in microwave and cooled to RT. After blocked with 3% H_2_O_2_ for 15 min, slides were incubated with 10% normal goat serum for 60 min at RT and then incubated with primary rabbit monoclonal antibody against mitochondrial SOD (SOD2, diluted to 1:400) overnight at 4°C. After washing with Tris-buffered saline, 0.1% Tween 20 (TBST) three times, sections were incubated at RT with an HRP-conjugated secondary antibody for 60 min. Proceeding washing with TBST three times, the localization of peroxidase conjugates were stained with DAB. The sections were photographed via NanoZoomer S360.

### Mitochondrial Isolation

Under the manufacturer’s instructions, fresh kidneys removed from the body within 1 h were used for mitochondrial isolation by tissue mitochondrial extraction kits (Beyotime, C3606). The mitochondria and cytoplasm were isolated by differential centrifugation method and mitochondria precipitations were kept in storage solution at 4°C for further western blot analysis.

### Western Blot Analysis

The above kidney mitochondria protein concentrations were detected by BCA Protein Assay kit and standards after being denatured for 10 min by heating at 95°C. Equal amount proteins (30 μg) were separated on 10% SDS-PAGE gels and transferred to polyvinylidene difluoride membranes. After being blocked with 5% BSA for 2 h at RT, the membranes were incubated with primary antibodies overnight at 4°C and washed three times with TBST buffer. The primary antibodies against SOD2, Cyt C, COX-IV were diluted to 1:1,000 by 3% nonfat milk prepared in TBST. Following the incubation of secondary antibody at RT, the protein signal was expressed through ECL substrate. The protein bands were visualized via chemiluminescence detection system (Bio-Rad, Hercules, CA, United States).

### Computed Tomographic Measurements

Two rats from the CI-AKI group and two from HXP group were initially anesthetized after the injection of iohexol (3 g iodine/kg body weight) and another two were form Ctrl group as control. Mediso nanoScan®SPECT/CT (Hungary) was used to detect iodine concentrations through scanning the whole rat body on the coronal plane and lower abdominal region through the horizontal section. Scan parameters (X-ray power was 70 kVp and 280 μA; exposure time was 300 ms) were set by two expertise radiologists remaining blinded to each group assignment and using region-of-interest (ROI) measurements. 300 s post-injection of iohexol was imaged and the computed tomographic (CT) signals expressed as Hounsfield unit (HU) were recorded. The images were processed and exported by InterView Fusion software.

### Transmission Electron Microscopy (TEM)

Fresh kidneys from different groups were harvested in 1 mm^3^, fixed in 2% glutaraldehyde in advance. Next, samples were dehydrated in ascending acetone series, embedded in Epon and polymerized. Each sample was dyed with uranyl acetate and lead citrate, the ultrastructures of the renal mitochondria were observed by the Hitachi H-7650 transmission electron microscope (Tokyo, Japan). Pathologists from outside were invited independently to quantify dysfunctional mitochondria in a blinded manner.

### Statistical Analysis

The software of Prism 8 (GraphPad) was employed to process the data. Qualitative data were shown as means ± standard error of the mean (SEM). Comparative of means were performed using a one-way analysis of variance (ANOVA) between multiple groups. Statistical significance was confirmed when *p* < 0.05.

## Result

### Circular Dichroism Spectral Analysis of Xylose-Pyrogallol Was Measured

**FIGURE 1:**
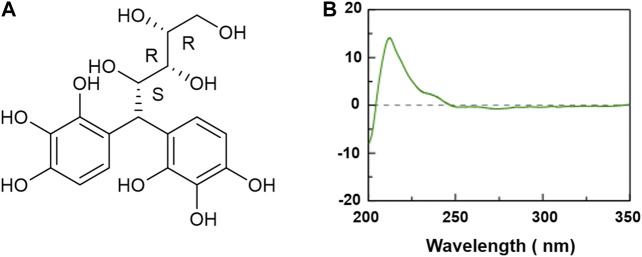
Chemical structure and circular dichroism spectra of Xylose-pyrogallol (XP) in water. **(A)** Chemical structure of XP. **(B)** CD spectra of XP in water at 0.5 mM.

The chemical structure of XP was shown in Figure 1A and its molecular weight was 384.33. The CD spectrum of XP was displayed in Figure 1B. As shown, XP had a positive Cotton effect at 212 nm and the absolute θ value was 16 medg. Since the chromophore of XP was the pyrogallol moiety, the configuration of the chiral carbon atom (C-4) near to the chromophore could change the Cotton effect by means of the empirical helicity rule (Kong and Wang, 2013).

### Xylose-Pyrogallol Pretreatment Improved Renal Function of Iohexol-Induced Renal Injury

**FIGURE 2:**
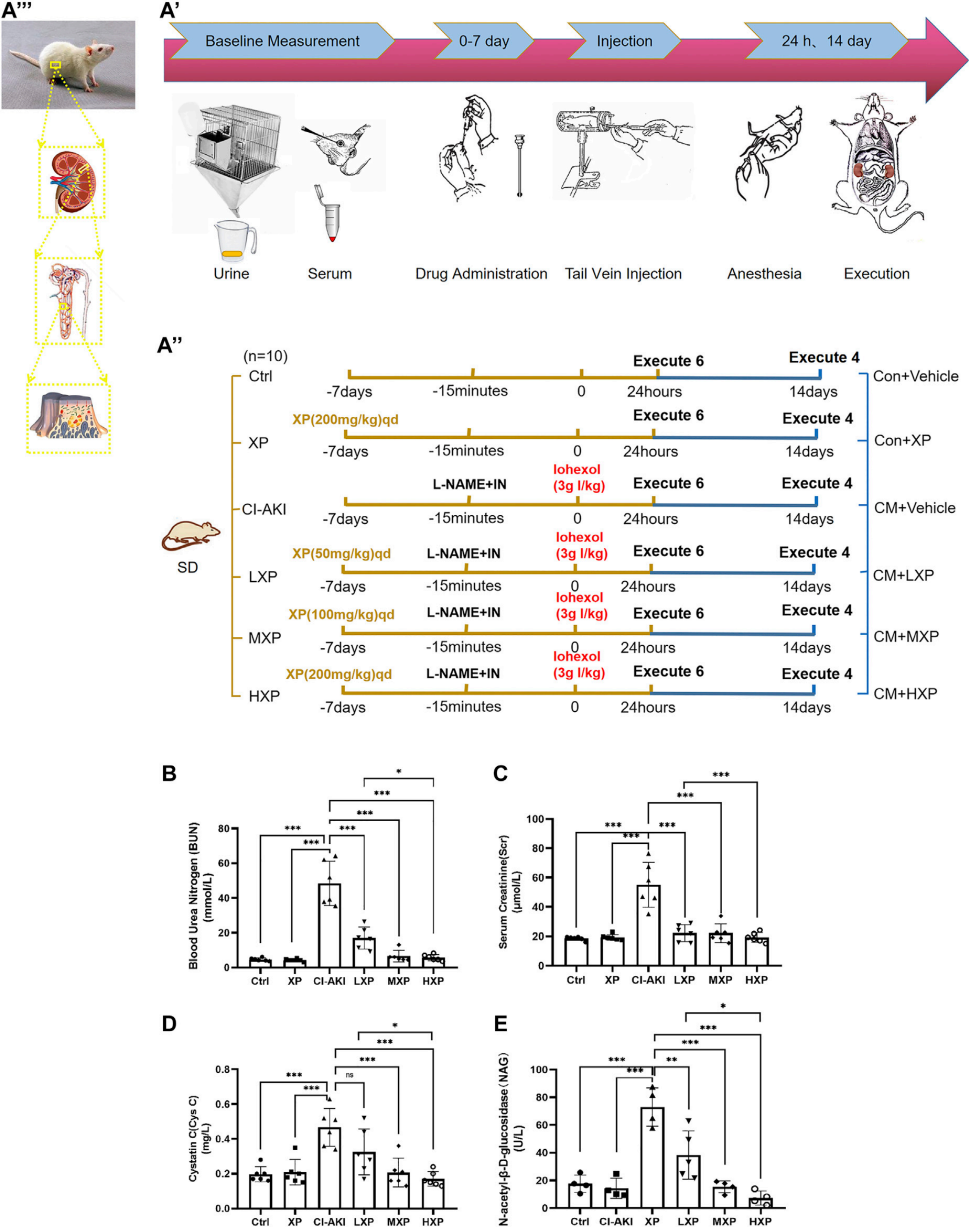
XP attenuates iohexol-induced nephrotoxicity in rats. **(A)** Diagrammatic representation of the inducible strategy in contrast-induced acute kidney injury (CI-AKI) rats. **(B)** Experimental process diagram ( Firstly, a total of 60 rats were randomly divided into six groups: shown as the left groups. The rats were administrated with or without XP orally for seven consecutive days before applying iohexol. Then CM was administrated to appropriate rats. 24 h after contrast administration, six animals of each group were chosen randomly and anesthetized, collected blood for confirmation of AKI, and kidneys removal for further experiments. 14 days after Iohexol, the left four rats were measured fibrosis degree. In order to distinguish the groups from the former, their names were changed as the right part). **(C)** experimental subject diagram. Iohexol caused renal dysfunction in the CI-AKI group, while the co-administration of XP decreased the level of BUN (B), Scr **(C)**, CysC **(D)** and uNAG **(E)**. Results are presented as mean ± SEM (*n* = 6 in each group). *p < 0.05; **p < 0.01; ***p < 0.001 based on one-way analysis of variance. Scr, serum creatinine; BUN, blood urea nitrogen; CysC, cystatin C; uNAG, urinary N-acetyl-β-D-glucosidase; Ctrl, control; CI-AKI, contrast induced-acute kidney injury; LXP, low dose XP combined with iohexol; MXP, medium dose XP combined with iohexol; HXP, high dose XP combined with iohexol.

As shown in Figure 2A, the whole experiment was performed as described and the disease model was built. The serum BUN, Scr, Cys C, and urinary NAG (uNAG) are considered significant markers of the relative severity of the renal injury. As shown in Figures 2B–E, the CI-AKI rats exhibited remarkable elevations in serum BUN, Scr, Cys C as well as uNAG compared with the Ctrl group (*p* < 0.001, Figures 2B–E), which indicated severe renal dysfunction in animals under vasoconstriction and iohexol-exposure conditions. Rats received XP alone exhibited no differences from those of control (*p* > 0.05), which generally indicated that no nephrotoxicity effect of XP was detected. Subsequently, we explored the effects of XP under CI-AKI conditions. Pretreatment with XP in iohexol-treated rats decreased the BUN, Scr, Cys C, and uNAG levels (*p* < 0.001). Moreover, XP at 200 mg/kg showed a better effect on protecting against iohexol-induced renal dysfunction in the dose-response experiment (*p* < 0.05 vs. the LXP group). Thus, XP alleviated AKI following the exposure to iohexol.

### Xylose-Pyrogallol Attenuated Morphological Alteration in Contrast-Induced Kidney Injury

**FIGURE 3:**
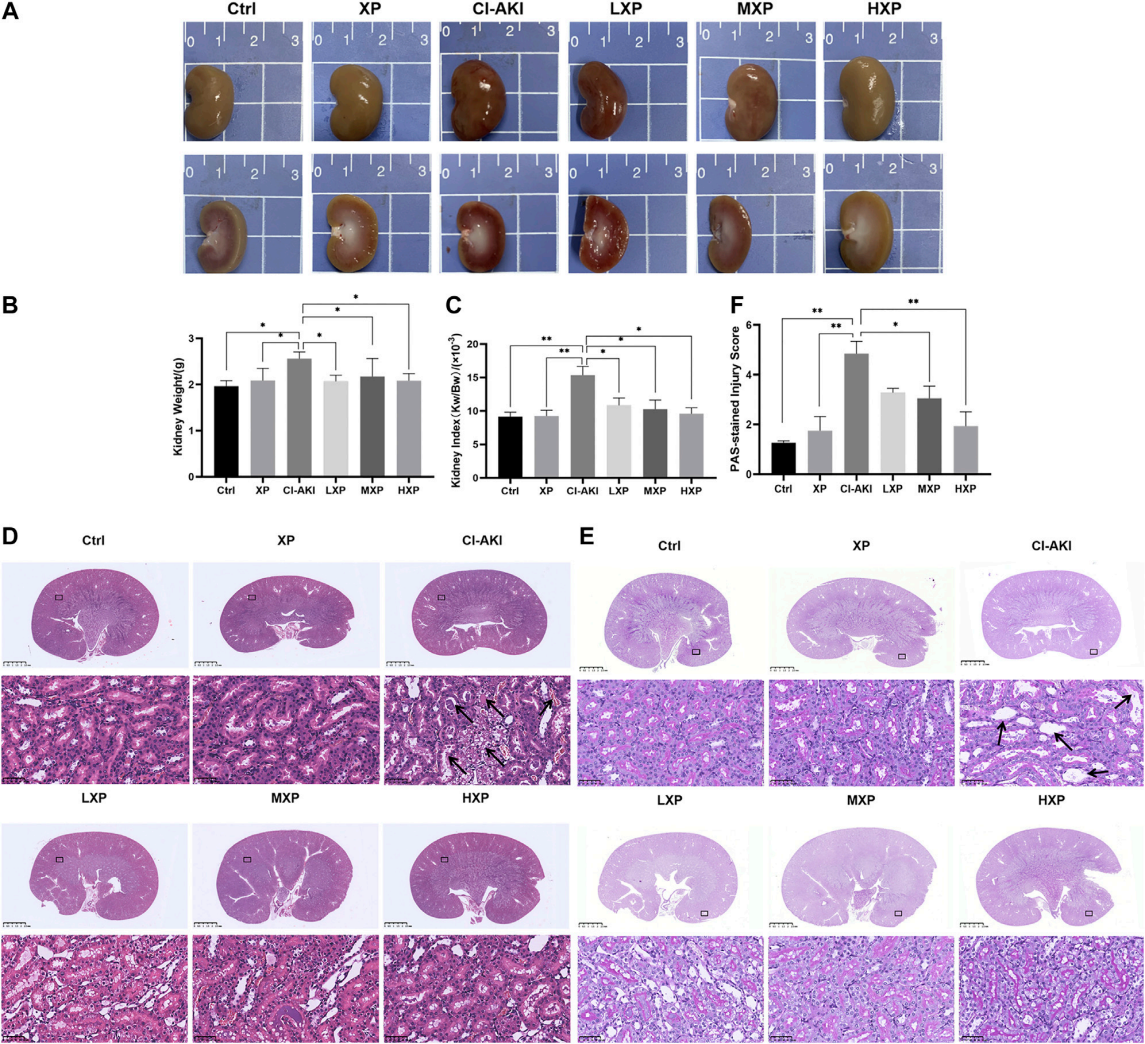
XP attenuated morphological damage in contrast-induced kidney injury. **(A)** Kidneys in group CI-AKI had obvious congestion than kidneys of other groups. **(B and C)** The kidney weight and KI were increased at 24 h after iohexol injection (*p* < 0.05). **(D)** Representative photomicrographs of HE-stained kidney sections were presented (Magnification:×1 and ×400, scale bars: 2.5 mm (upper) and 50 μm (lower)). **(E)** PAS-stained kidney sections (Magnification:×1 and ×400, scale bars: 2.5 mm (upper) and 50 μm (lower)) and **(F)** semi-quantitative scoring analysis of tubular degeneration were presented. Marked plenty of tubular vacuolization and degeneration with shedding cells in the lumen, congestion in the vein, and obscure cell boundaries was shown in CI-AKI. For the semi-quantitative analysis of morphological changes, the extent of tubular injury was graded with a score from 0 to 4 points. Data were presented as mean ± SEM (*n* = 6 in each group).*p < 0.05; **p < 0.01; ***p < 0.001 based on one-way analysis of variance. Representative images are presented. HE, hematoxylin and eosin; PAS, Periodic Acid-Schiff; KI, kidney index; Ctrl, control; CI-AKI, contrast induced-acute kidney injury; LXP, low dose XP combined with iohexol; MXP, medium dose XP combined with iohexol; HXP, high dose XP combined with iohexol.

As shown in Figure 3A, the rat kidneys of CI-AKI group were swollen and enlarged in the gross pathology. Besides, obvious congestion zones can be seen at the cortico-medullary junction in the coronal plane when compared with other groups. The control renal had a clear junction of the cortex and medulla without congestion and swelling. Moreover, kidney weight and kidney index (KI), which is kidney weight to body weight ratios (Kw/Bw), were escalated in the CI-AKI group (*p* < 0.05, Figures 3B,C). As depicted in Figure 3D, the CI-AKI group rats by HE staining pictured plenty of tubular vacuolization and degeneration with shedding cells in the ducts, congestion in the vein, and obscure cell boundaries (indicated by black arrowheads). Yet, when XP was gavaged beforehand to contrast administrated rats, a reduction of renal tubules lesions was observed. As pathological parameters were shown in Table 1, the CI-AKI group received the highest total scores among all groups, and rats in the treatment groups (XP plus CM treated groups) received significantly lower pathological scores. In agreement with physiological alteration in HE-staining, the CI-AKI group animals received the highest total scores under PAS-staining pathological evaluation with disappeared brush edges (indicated with black arrowheads) and tubular vacuolization (Figure 3E), and rats in the LXP, MXP, HXP groups received lower pathological scores (*p* < 0.05, Figure 3F). The most severe damages were all observed in the renal cortico-medullary boundary zone whereas XP effectively reversed the iohexol-damage situation.

**TABLE 1:** Histopathological changes in kidneys.

Tubular injury score	− or 0	± or 1	+ or 2	++ or 3	+++ or 4
Ctrl (*n* = 6)	60	0	0	0	0
	(0.00 ± 0.00)				
CI-AKI (*n* = 6)	0	0	6	24	30
	(3.4 ± 0.70^a^)				
XP (*n* = 6)	54	6	0	0	0
	(0.1 ± 0.32)				
LXP (*n* = 6)	0	18	24	18	0
	(2.0 ± 0.82^a^ ^,^ ^b^)				
MXP (n = 6)	30	18	6	6	0
	(0.8 ± 1.03^a^ ^,^ ^b^)				
HXP (*n* = 6)	42	18	0	0	0
	(0.3 ± 0.48^a^ ^,^ ^b^ ^,^ ^c^)				

Descriptions of the groups are in the text. Ten consecutive (×400) fields of cortex and outer stripe of medulla were selected randomly from each sections in each group (60 fields in each group).

a *p* < 0.05 compared with Ctrl.

b *p* < 0.05 compared with CI-AKI.

c *p* < 0.05 compared with LXP.

### Xylose-Pyrogallol Reduced Iohexol-Induced Apoptosis in Rats

**FIGURE 4:**
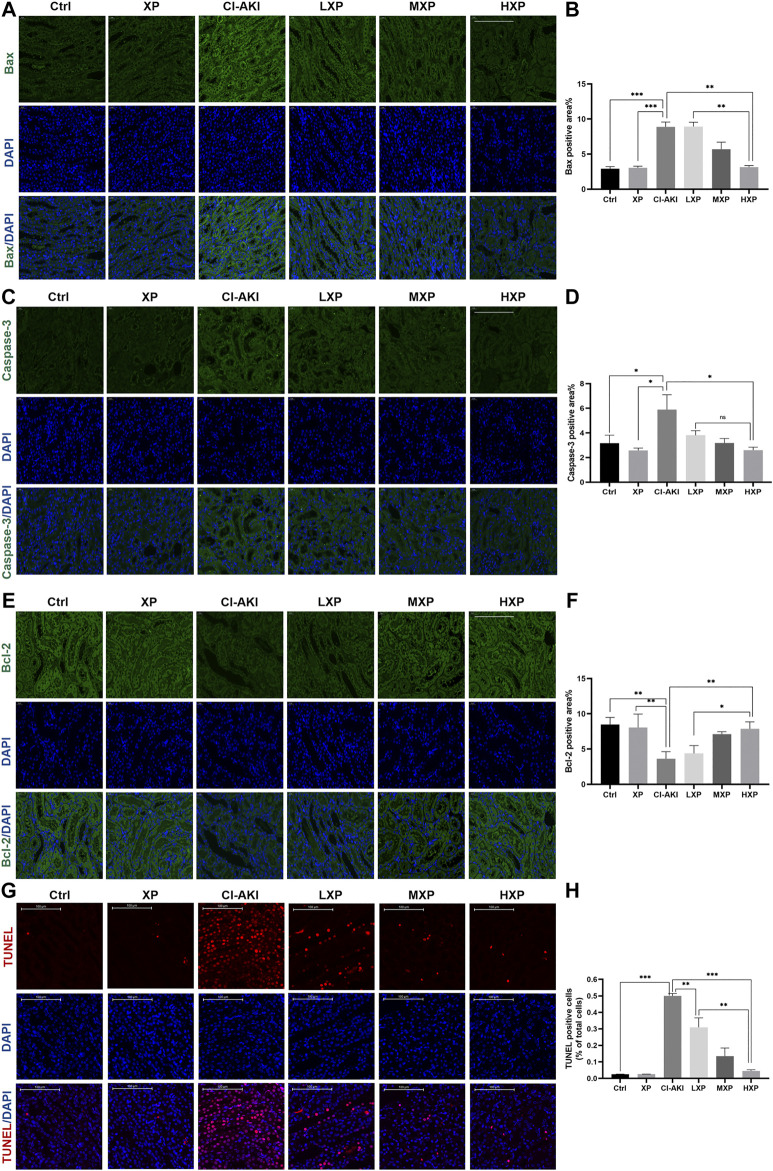
The anti-apoptotic effects of XP in CI-AKI**. (A)** Bax (green) signals (Magnification: ×400, scale bar: 100 μm) and **(C)** caspase-3 (green) signals (Magnification: ×400, scale bar: 100 μm) were immunofluorescent analyzed. The results indicated that iohexol induced obvious apoptosis in the kidney. Decreased signals on bax and caspase-3 were detected when XP was given to iohexol-injured rats. **(B and D)** The semi-quantitive analysis showed the bax, caspase-3 positive percentage in each group. **(E)** Bcl-2 (green) signals (Magnification: ×400, scale bar: 100 μm) increased dose-dependently in treatment groups whereas the signal in the CI-AKI group was weak, which demonstrated that XP can mitigate iohexol-induced apoptosis. **(F)** Columns showed the bcl-2 positive percentage in each group. **(G)** TdT-mediated dUTP nick end labeling (TUNEL) staining (red) of kidney sections (Magnification: ×400, scale bar: 100 μm). Iohexol exposure induced significantly increased renal apoptosis. XP obviously and dose-dependently alleviated iohexol-induced kidney tubular apoptosis. **(H)** Columns showed the apoptotic positive cells percentage in each group. Data are presented as mean ± SEM (*n* = 6 in each group). *p < 0.05; **p < 0.01; ***p < 0.001 based on one-way analysis of variance. Representative images are presented. TUNEL, TdT-mediated dUTP nick end labeling; Ctrl, control; CI-AKI, contrast induced-acute kidney injury; LXP, low dose XP combined with iohexol; MXP, medium dose XP combined with iohexol; HXP, high dose XP combined with iohexol.

In order to investigate the mechanisms of the renoprotective effects of XP in rats under vasoconstriction condition with CI-AKI, we examined signaling proteins expression levels of three apoptosis markers: caspase-3 and other two bcl family proteins, bcl-2 and bax in cortex regions of kidney (Figures 4A,C,E), as these proteins were well known participating in execution of cellular apoptosis. Contrast with the Ctrl and XP groups kidneys with minor amounts of bax and caspase-3 being expressed, intense signals of bax (*p* < 0.001, Figure 4B) and caspase-3 (*p* < 0.05, Figure 4D) were observed at the proximal tubule in CI-AKI group. The apparent reduction of bax (*p* < 0.001, Figure 4B) and caspase-3 (*p* < 0.05, Figure 4D) expression levels were observed when XP was given to iohexol-injured animals. Moreover, bcl-2 was anti-apoptotic proteins so that bcl-2 protein expressed the opposite experiment result (Figure 4E), significant elevation of bcl-2 expression was analyzed in HXP group (*p* < 0.01, Figure 4F), indicating that XP significantly changed iohexol-induced caspase family and bcl family-associated apoptosis in kidneys, despite the fact that no apparent differences among different-dose treatment groups in expression levels of caspase-3 (*p* > 0.05, Figure 4D). In agreement with immunofluorescent assay study, TUNEL assay kit was used to assess the expression levels of apoptotic positive cells, indicated a weak TUNEL-positive signal in the Ctrl and XP groups kidneys in cortex regions, but a high proportion of positive cells were detected in the CI-AKI group (*p* < 0.001, Figures 4G,H). More importantly, when XP was given to iohexol-administrated rats, the enhanced expression of TUNEL-positive cells was dampened in kidney sections.

### Xylose-Pyrogallol Maintained Cellular Localization of Mitochondrial Enzyme Cytochrome c in Kidneys

**FIGURE 5:**
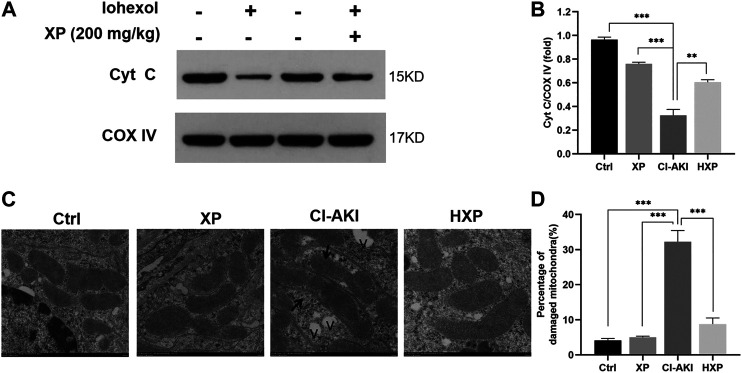
Mitochondrial enzyme cytochrome c (Cyt C) assessments in kidneys. **(A)** Western-blotting analysis showed the majority of the Cyt C protein appeared in the mitochondria fraction of Ctrl, XP and HXP groups. However, a significant reduction of total Cyt C protein expression in the mitochondria fraction was observed in iohexol-induced animals. **(B)** Densitometric analysis (protein quantification) of western blotting signals using image J software. Fold change in each protein level normalized to COX IV is shown numerically. **(C)** Mitochondrial ultrastructure of the kidney tissues were scanned using Transmission electron microscopy (TEM) (Scale bar: 500 nm). “v” indicated vacuolization. **(D)** Data were shown as a plot of the percentage of damaged mitochondria from 10 images of each group. Data are presented as mean ± SEM (*n* = 6 in each group). *p < 0.05; **p < 0.01; ***p < 0.001 based on one-way analysis of variance. Representative images are presented. TUNEL, Cyt C, cytochrome c; COX IV, cytochrome c oxidase IV, TEM, transmission electron microscope; Ctrl, control; CI-AKI, contrast induced-acute kidney injury; HXP, high dose XP combined with iohexol.

In the above experiments including dose-response, XP at 200 mg/kg showed a better effect on protecting against CI-AKI (Figures 2–4). Thus, the HXP group representing treatment groups was involved in the next experiments exploring mechanisms associated with the CI-AKI pathogenesis and the renoprotective effects of XP. It can be seen from the known result that intrinsic cell apoptosis was proved involving in the iohexol-induced damages and it was triggered by damaged mitochondria with delocalized Cyt C (Liu et al., 2019), thus we next detected Cyt C content in mitochondrial protein, an internal mitochondria membrane protein, which is essential to mitochondria redox reaction and has been confirmed to launch caspase-dependent apoptosis upon Cyt C releasing out of mitochondria (Elena-Real et al., 2018). COX IV was used as an internal reference in mitochondria. As showed in Figure 5A, we detected that a minimal Cyt C was expressed in mitochondria fraction in the CI-AKI group. However, in the Ctrl and XP groups, Cyt C still remained in the mitochondria fraction (*p* < 0.05, Figure 5B). Moreover, a significant recovery on mitochondrial Cyt C was detected in the mitochondria fraction in the HXP group (*p* < 0.05, Figure 5B), indicating that XP attenuated the release of Cyt C from mitochondria to cytoplasm induced by iohexol.

### Xylose-Pyrogallol Improved Mitochondrial Ultrastructure in Renal

To observe the effects of iohexol on mitochondrial ultrastructure more objectively, we applied TEM and as pictured in Figure 5C, normal mitochondria with membrane was intact, and few lysosomes and autophagosomes were presented in Ctrl and XP groups. However, a large amount of mitochondria with irregular morphologies, somewhat fragmented and swollen mitochondria, loss of mitochondrial crests, and even vacuolization (indicated with “v”) in the matrix were evident in the CI-AKI group. In Contrast to the CI-AKI group, XP alleviated the damage of mitochondrial ultrastructure induced by iohexol exposure because most of the mitochondrial membrane was intact, the inner ridge was clear, and arranged neatly in the HXP group. Moreover, the quantitative analysis implied that the percentage of damaged mitochondria in the HXP group was obviously less than the CI-AKI group (*p* < 0.001, Figure 5D).

### Xylose-Pyrogallol Treatment Attenuated Iohexol-Induced Mitochondrial Redox Disorder in Rats

**FIGURE 6:**
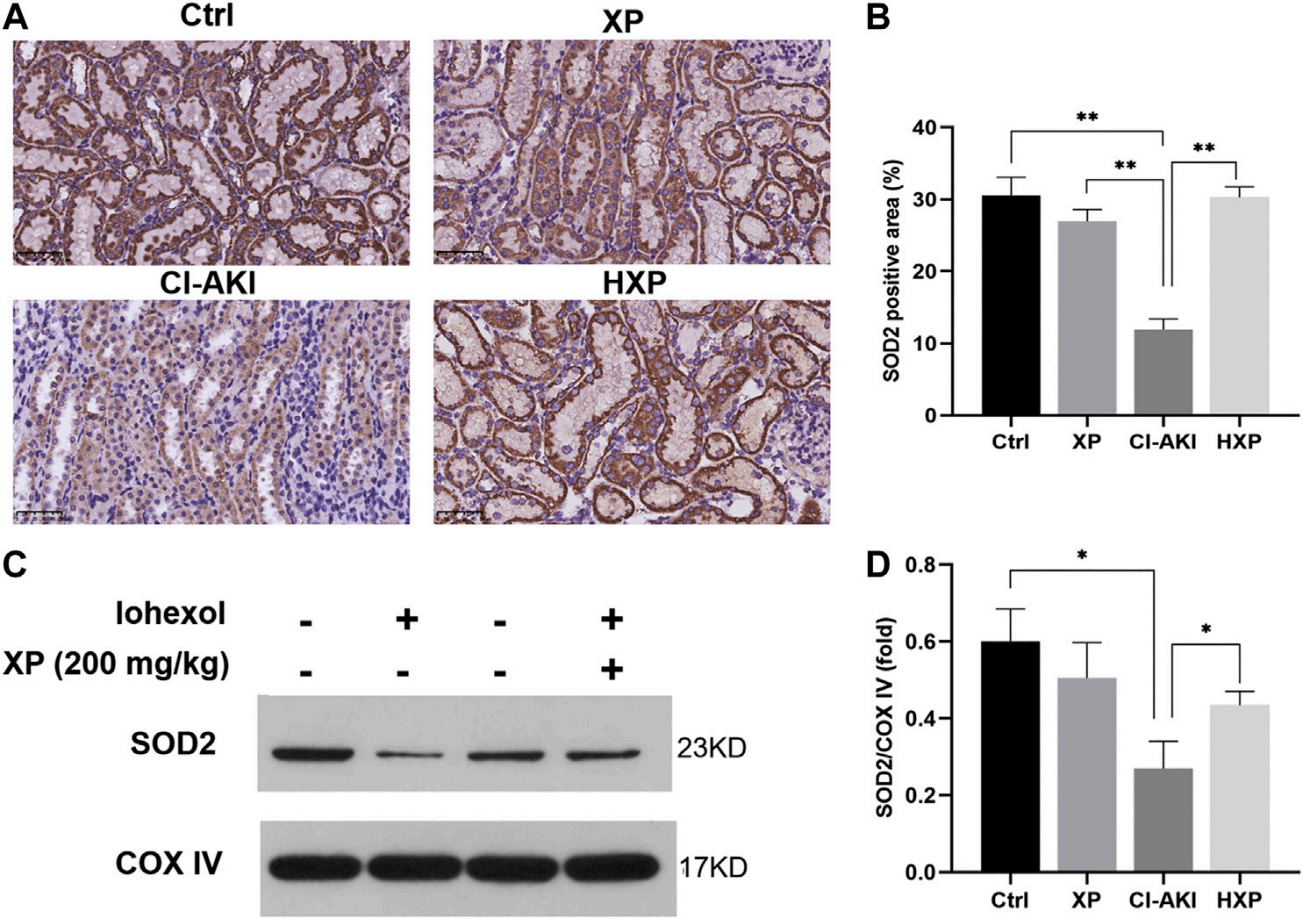
XP treatment attenuated iohexol-induced mitochondrial dysfunction in rats. **(A)** Immunohistochemistry staining of superoxide dismutase 2 (SOD2) in renal tissues (Magnification: × 400, Scle bars: 50 μm). **(B)** The quantification of SOD2 in the four groups. **(C)** Western blotting analysis of SOD2 in four groups of renal tissues. COX IV was used as a loading control. **(D)** Densitometric analysis (protein quantification) of western blotting signals using image J software. Fold change in each protein level normalized to COX IV is shown numerically. Data are presented as mean ± SEM (*n* = 6 in each group). *p < 0.05; **p < 0.01; ***p < 0.001 based on one-way analysis of variance. Representative images are presented. SOD2, superoxide dismutase two; COX IV, cytochrome c oxidase IV; Ctrl, control; CI-AKI, contrast induced-acute kidney injury; LXP, low dose XP combined with iohexol; MXP, medium dose XP combined with iohexol; HXP, high dose XP combined with iohexol.

Mitochondrial structural damages result in the generation of mitochondrial reactive oxygen species (ROS) during the usage of oxygen in the cellular respiratory chain. To further clarify the role of XP in modulating mitochondrial redox balance to maintain mitochondrial function in kidneys, we examined the protein levels of SOD2 in kidneys of iohexol-treated rats. SOD2 played a vital role in regulating the concentration of ROS in mitochondria, and it was encoded by the SOD2 gene and bound to the superoxide by-products of oxidative phosphorylation and converted them into hydrogen peroxide and oxygen. As shown by the results of immunohistochemistry staining (Figure 6A), the protein levels of SOD2 were down-regulated significantly by iohexol in the CI-AKI group, which were remarkably restored by XP (200 mg/kg) treatment with increased positive expression in the cytoplasm (*p* < 0.05, Figure 6B). Similarly, the Western blotting analysis showed the protein levels of SOD2 also up-regulated after XP treatment as compared with the CI-AKI group (Figures 6C,D).

### Xylose-Pyrogallol Pretreatment Protected Against Iohexol-Induced Oxidative Stress in Kidneys

**FIGURE 7:**
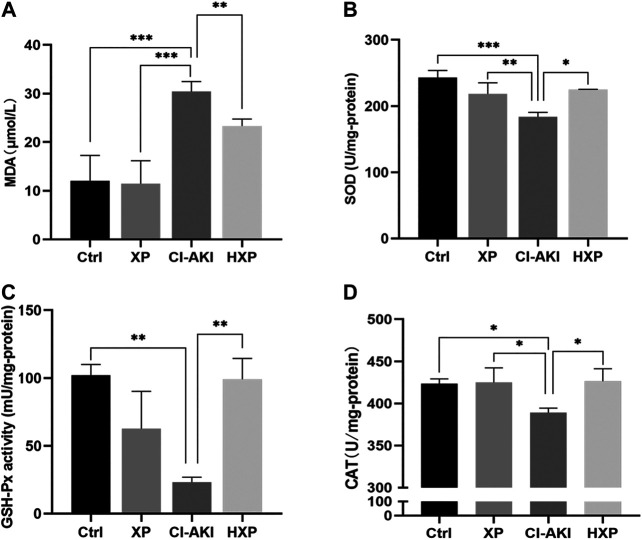
Inhibition of iohexol-induced oxidative stress by XP pretreatment. **(A)** The amount of malondialdehyde (MDA) was measured in the four groups. **(B)** SOD levels were measured in the four groups. **(C)** GSH-Px levels were measured in the four groups. **(D)** CAT levels were measured in the four groups. An increase of MDA and a reduction of SOD, GSH-Px and CAT were detected in iohexol-injured kidneys, which were remarkably restored in the HXP group. Data are presented as mean ± SEM (*n* = 6 in each group). *p < 0.05; **p < 0.01; ***p < 0.001 based on one-way analysis of variance. MDA, malondialdehyde; SOD, superoxide dismutase; GSH-Px, Glutathione peroxidase;CAT, catalase; Ctrl, control; CI-AKI, contrast induced-acute kidney injury; LXP, low dose XP combined with iohexol; MXP, medium dose XP combined with iohexol; HXP, high dose XP combined with iohexol.

Oxidative stress was thought to be the primary reasons for contrast-mediated renal function deterioration (Scharnweber et al., 2017b). For the purpose of verifying the effects of XP on CM-induced renal oxidative stress, the levels of antioxidant system in kidneys were experimented. As shown in Figure 7A, contrast injection lead to significantly increased production of MDA in kidney homogenate compared with the Ctrl group (*p* < 0.001). However, pretreatment with XP was able to efficiently inhibit the generation of lipid peroxidation and reduce MDA content compared with the CI-AKI group (*p* < 0.01). Besides, compared with the Ctrl, CM injection markedly decreased the activities of SOD (*p* < 0.05, Figure 7B), GSH-Px (*p* < 0.01, Figure 7C) and CAT (*p* < 0.05, Figure 7D) in renal, and XP pretreatment could significantly up-regulate the level of SOD (*p* < 0.01, Figure 7B), GSH-Px (*p* < 0.01, Figure 7C), and CAT (*p* < 0.05, Figure 7D) in renal tissues compared with the CI-AKI group.

### Xylose-Pyrogallol Increased the Body Weight and Attenuated Iohexol-Induced Long-Term Generation of Renal Fibrosis

**FIGURE 8:**
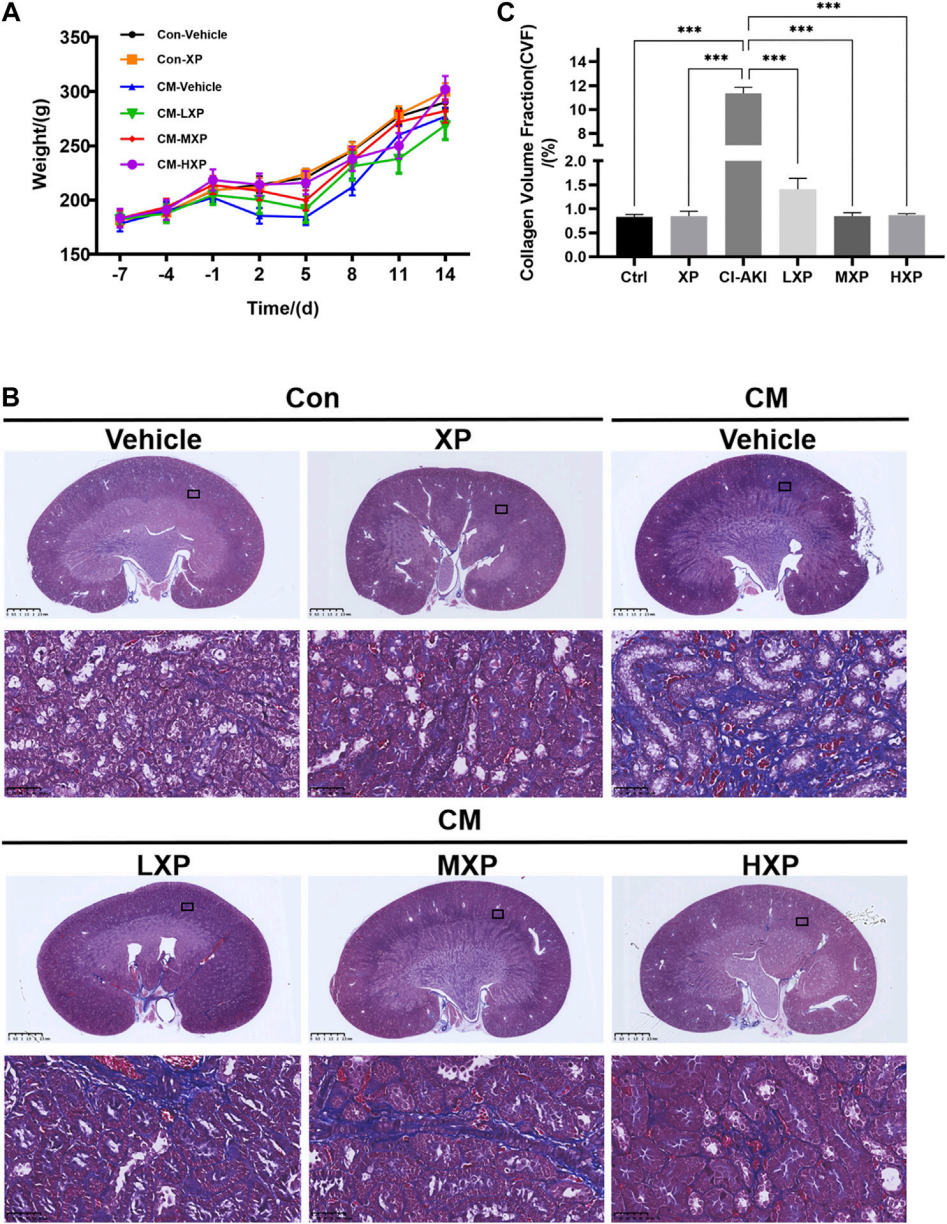
Body weight and Masson's trichrome staining. **(A)** The rats weight changes with or without treatment with XP during 21 days. **(B)** Masson’s trichrome staining (Magnification: × 400, scale bar: 50 μm) showed an increased fibrosis stained in blue in iohexol-receiving animals while significantly less collagen fiber deposition was observed in Con-Vehicle group and CM-LXP, CM-MXP, CM-HXP groups. Images presented are representative images. **(C)** The quantification of Masson’s trichrome staining was presented as collagen volume fraction (CVF) values. Data are presented as mean ± SEM (*n* = 4 in each group). *p < 0.05; **p < 0.01; ***p < 0.001 based on one-way analysis of variance. CVF, collagen volume fraction; Con, control; CM, contrast medium; LXP, low dose XP; MXP, medium dose XP; HXP, high dose XP.

CI-AKI is not only a short-lived phenomenon and acute disease, but also has been proven to be associated with long-term adverse consequences. Chronic kidney disease (CKD) with renal fibrosis after AKI may explain the poor long-term prognosis of CI-AKI (Scharnweber et al., 2017). Thus a 14-days observational experiment (*n* = 4) was carried out after building the CI-AKI model. The body weight was recorded every day during the experiment and and Masson’s trichrome staining was proceeded. Besides, the markers of renal dysfunction and oxidative stress were measured to further evaluate the statue of rats at the 14-days time point (Supplementary Figures S1,S2). As shown in Figure 8A, the mean body weight of the Con-Vehicle group increased over time. Although the mean body weights in six groups reached the normal value at the end of the observational experiment, the body weights of rats decreased in a period of five days after iohexol injection, which was improved by XP. Beyond that, the CM-HXP group has the most similar body weight growth curve with the Con-Vehicle group. It was interesting to note that the indicators of serum renal function in the CM-Vehicle group recovered to the nearly normal condition 14 days after CM injection (*p* > 0.05 Supplementary Figure S1) and the oxidative stress status were not as intense as before (Supplementary Figure S2). But the traces of long-term injury caused by iohexol were manifested in kidney pathological sections and XP effectively protected CM-HXP group rats from serious renal fibrosis. Masson’s trichrome stain indicated an obviously higher amount of fibrosis in CM rats (Figure 8B, blue indicated collagen fibers). In spite of an increase of fibrosis in the treatment groups, the amount was markedly less when compared with the CM-Vehicle group (*p* < 0.001, Figure 8C). Taken together, the data indicated that XP ameliorated iohexol-caused body weight decrease and renal interstitial fibrosis.

### The Effect of Xylose-Pyrogallol on the Imaging of Iohexol Was Evaluated by Computed Tomographic Measurements

**FIGURE 9:**
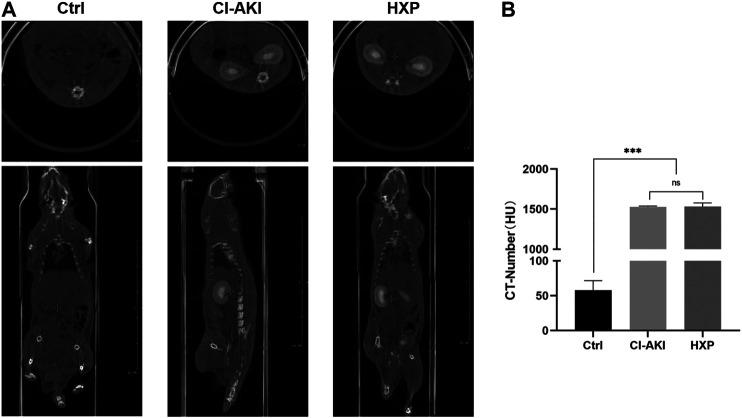
The effect of XP on the imaging of iohexol **(A)** The enhanced CT images showed pronounced iodine exposure in the entire kidney after the injection of iohexol and plain CT scan in control rats at 200 s in the coronal plane and cross-section respectively. **(B)** The CT-number of three groups. Images presented are representative images. Data are presented as mean ± SEM (*n* = 2 in each group). ^ns^ *p* > 0.05; *p < 0.05; **p < 0.01; ***p < 0.001 based on one-way analysis of variance. CVF, collagen volume fraction; Ctrl, control; CI-AKI, contrast induced-acute kidney injury; HXP, high dose XP combined with iohexol.

Iohexol is a sort of low-osmolality iodinated contrast media which is widely used in clinical diagnosis and treatment. Therefore, we evaluated the XP effect on the imaging activity of iohexol. After the administration of iohexol (3 g iodine/kg) based on 12-h dehydration in rats, we recorded CT-numbers of the iohexol-administrated rats and iohexol plus XP (200 mg/kg)-administrated rats (*n* = 2 in each group) at 200 s after injection of contrast medium. At this point, the CT value measured for iohexol was 1,533.6 ± 248 HU. For iohexol plus XP, the HU values was increased to 1,580.3 ± 523 HU, and there are no significant differences between them (*p* > 0.05, Figures 9A,B). However, the CT signal detected in the control rats was markedly lower than the other two groups (*p* < 0.001, Figure 9B). All these data suggested that XP might not diminish the imaging effect of the contrast medium.

## Discussion

CI-AKI is one of the main causes of hospital-acquired AKI (Takahashi et al., 2017), and it is generally acknowledged as animal model to explore the efficiency of protective agents. Iohexol is our choice of a radiocontrast agent, a low-osmolar nonionic dimeric iodinated contrast medium, also reflects common clinical practice. It is widely accepted that in individuals with normal renal function, the risk of CI-AKI is negligible, pre-existing renal disease and other common basic diseases are its greatest independent risk factors (Windpessl and Kronbichler, 2018). Thus vasoconstriction condition was achieved by injection of indomethacin and L-NAME before iohexol administration. In our study, treatment with iohexol based on vasoconstriction disrupted renal morphological structure, and Scr increased >50% from baseline, demonstrating that the CI-AKI model had been generated successfully. Until now, there are no widely accepted strategies that are effective in the protection of CI-AKI except intravenous hydration with normal saline (Nijssen et al., 2017). Developing nephroprotective agents is thus crucial to CI-AKI in high-risk patients. Nowadays attention has been paid to discover compounds of natural products in preventing renal tubular cell injury in AKI (Baradaran et al., 2015; Ma et al., 2015). Moreover, AKI is also an important pathogenic factor in the development and progression of CKD (Ozkok and Edelstein, 2014; Guo et al., 2018). In present research, the effect of an natural product analog was evaluated on CI-AKI and iohexol-induced long-term injury, and we aimed to explored the underlying mechanisms in rats involving complex pathway crosstalk related to oxidative stress, mitochondrial dysfunction, as well as apoptosis.

The circulating levels of metabolites BUN, Scr, and Cys C are recognized as classic biochemical markers for assessing renal dysfunction. In our study, Co-injection of indomethacin, L-NAME, and iohexol successively can significantly increase the serum levels of BUN, Scr, and Cys C in rats, which were significantly ameliorated by the XP pretreatment. The filtration and transport mechanisms in the proximal tubule epithelia render the kidney highly susceptible to toxicant-induced renal injury (Inoue et al., 1999). For instance, one of the most sensitive biomarkers of cell injury to the proximal tubules is the excretion of uNAG (Inoue et al., 1999). Identical to the results of serum biomarkers, the uNAG results revealed that XP possessed nephroprotective effect as well. Moreover, results of histopathology also implied that XP could reduce necrosis of tubular epithelial cells, vacuolization, and loss of brush borders induced by iohexol in a dose-dependent manner. Therefore, we concluded that XP exerted a significant nephroprotective effect on CI-AKI.

As it well known, apoptosis refers to one of the most common programmed cell death, and a self-protecting mechanism of elimination of the unwanted or abnormal cells inside the body (Inoue et al., 1999). This natural process can be regulated by the activation of the intrinsic, mitochondrial-dependent pathway, and the extrinsic, death receptor-mediated pathway (Kang et al., 2012). The intrinsic pathway is triggered by various intracellular and extracellular stresses whose signals converge to mitochondria in the end (Su et al., 2014). Many studies have confirmed that mitochondrial damage displays a pivotal role in apoptosis (Quintavalle et al., 2011). Mitochondria is one of the most complex organelles with a variety of important functions such as providing energy and maintaining homeostasis of its own, which is essential for kidney function (Ishimoto and Inagi, 2016). Kidneys require a large amount of energy because they reabsorb 99% of ultrafiltrate through glomeruli to keep the body in homeostasis. Tubular cells have plentiful mitochondria, and recent studies attach importance to the role of mitochondrial damage in the progression of kidney disease (Ishimoto and Inagi, 2016; Maekawa et al., 2019). Actually, previous researches have proved that tubular damage of AKI induced by cisplatin (Ortega-Dominguez et al., 2017) and ischemia/reperfusion (Hall et al., 2013) is correlative with mitochondrial alterations. It has been acknowledged that bcl-2 family proteins play the most important role in the earlier stage of apoptosis. The interaction of bcl-2 family members can trigger the loss of mitochondrial membrane potential and mitochondrial outer membrane permeabilization, and in turn cause mitochondrial damage. The disruption of mitochondrial membrane potential can lead to the release of Cyt C from mitochondria into cytosol and the activation of caspase cascade in the mitochondrial pathway. Caspases are a family of cysteine proteases responsible for apoptosis in mammalian cells and caspase-3 is an important executor caspase of apoptosis (Gao et al., 2020). Thus, the balance between the pro-survival member bcl-2 and the pro-apoptotic member bax plays an important role in the regulation and execution of intrinsic apoptosis (Singh et al., 2019). Moreover, the activation of caspases-3 is necessary in the death receptor pathway or mitochondrial pathway (Elmore, 2007). Thus, the expression levels of bax, caspase-3, bcl-2 and Cyt C were detected in this study. Consistent with previous western blotting results (Elmore, 2007), our immunofluorescence analysis revealed that the expression of bax and caspase-3 was increased while the expression of bcl-2 was decreased in the CI-AKI group. The western blotting analysis indicated that mitochondrial Cyt C protein was reduced in the CI-AKI group. However, these alterations of protein expression were restored by XP pretreatment. Besides that, TUNEL staining was used to characterize the apoptotic response and locate the apoptotic cell in renal sections, which indicated the degradation of DNA molecules by apoptotic enzymes in the end (Li et al., 2018). Consistent with previous research (Tan et al., 2017), contrast injection significantly increased the number of apoptosis-positive cells. However, the number of apoptotic cells in XP pretreatment groups was significantly reduced compared with the CI-AKI group. That effect was consistent with the improvement of serum biomarkers and kidney histopathology, which further demonstrated that XP can protect renal tissues from CI-AKI by regulating apoptosis pathways, which included inhibition of end-stage execution of apoptosis as well as maintaining the balance of mitochondrial homeostasis.

Oxidative stress has been widely regarded as one of the most significant mechanisms in the pathogenesis of CI-AKI as well as a potent pro-apoptotic factor (Detrenis et al., 2005). Meanwhile, XP is one analog of EGCG with strong antioxidant function. Previous studies clearly demonstrated that iodinated contrast media administration enhanced oxidative stress and the excessive production of ROS within the kidney (Zager et al., 2003; Toprak et al., 2008; Kongkham et al., 2013). ROS are mainly produced by biochemical reactions in the mitochondria when they are damaged, and leakage of electrons from the mitochondrial electron transport chain is the main source of mitochondrial ROS. In this research, mitochondrial damage ultrastructures were observed through TEM. As reported, ROS accumulated via mitochondrial dysfunction and increasing oxygen consumption, and it may exert direct tubular and endothelial injury, and further intensify renal dysfunction of mitochondria and parenchymal hypoxia by virtue of endothelial dysfunction and dysregulation of tubular transport (Andreucci et al., 2014; Kiyonaga et al., 2020). One of the serious damage caused by ROS is attacking polyunsaturated fatty acids on cell membranes, causing them to oxidize and form lipids peroxidation (Tian et al., 2012). MDA is one of the major lipid peroxidation products, which is the most representative indicator of oxidative damage in the body nowadays, for it connects with proteins, enzymes, or receptors embedded on the cell membrane to change its function and eventually lead to cell damage and even death (Ho et al., 2013; Nourazarian et al., 2014). The production of free radicals also can be blocked by endogenous antioxidant systems such as antioxidant metalloenzyme SOD, GSH-Px, and CAT enzymes (Toprak et al., 2008; Kongkham et al., 2013). According to the different metal prosthetic groups, SOD can be roughly divided into three categories, namely Cu/ZnSOD, MnSOD (also known as SOD2), and FeSOD. Cu/Zn-SOD mainly exists in the cytoplasm while SOD2 is widely presented in the mitochondria (Dehghani et al., 2020), and the reduction of its expression and activity will weaken the scavenging effect of superoxide ions (a form of ROS) produced in the mitochondria, leading to peroxidative damage of mitochondrial DNA. In addition, GSH-Px is an important peroxidase that exists widely in the body. It can catalyze GSH to GSSG and reduce toxic peroxides to non-toxic hydroxyl compounds, thereby protecting the structure and function of cell membranes from the damage of peroxides (Zeng et al., 2008). In our study, we observed in the CI-AKI group that mitochondrial ultrastructure was damaged and mitochondrial ROS excessively generated, which proved by the depletion of mitochondrial SOD2 in western blot and immunohistochemistry. In the oxidative stress aspect, MDA was increased and antioxidant enzyme activities of SOD, GSH-Px, CAT were reduced in the renal tissues exposed to iohexol, which reflected an augmentation of lipid peroxidation and a diminution of antioxidation, but all of these found perturbations in the antioxidant system could be restored by the pretreatment of XP. Therefore, oxidative stress induced by iohexol is a relative excess of oxidants caused by increased free radical production and decreased antioxidant defense systems. Besides, previous study has suggested that the reduction of oxidative stress is also likely achieved by maintaining Cyt C at the inner mitochondria membrane and thus improves mitochondria redox ability and mitochondria integrity (Liu et al., 2019). Our results indicated that pretreatment with XP could protect against CI-AKI in rats by ameliorating mitochondrial morphological damages as well as mitochondrial and systemical oxidative stress in kidneys.

AKI is a risk factor for CKD, which is characterized by renal interstitial fibrosis. Over the past decade, multiple studies have shown a strong epidemiological link between AKI and the subsequent development of CKD (Ronco et al., 2019). Several mechanisms such as mitochondrial bioenergetics alterations and oxidative stress imbalance have been proposed that once it was triggered, it could contribute to the development of CKD (Granata et al., 2009; Che et al., 2014; Aparicio-Trejo et al., 2017). In fact, impairment of mitochondrial bioenergetics and dynamics have been reported in the early stage of CKD model experiments (Granata et al., 2009; Che et al., 2014; Aparicio-Trejo et al., 2017), which verified that there were relations in terms of mechanism between AKI and CKD. In the present research, mitochondrial changes and oxidative stress imbalance were described as above, and interstitial fibrosis have been presented in renal sections in the CM-Vehicle group two weeks later, and an obvious reduction of collagen fibers was shown in XP pretreatment groups, which suggested that the application of XP effectively delayed the progression of AKI-based CKD. Noteworthily, the serum markers and stress states would experience a recovery spontaneously a few days after CI-AKI while kidney injury may be progressing. In fact, during the clinical practice, the patients with slight serum markers of renal dysfunction and sever result in kidney biopsy do exist and they usually undergo poor prognosis. In the present study, we also evaluated whether XP influenced the imaging effectiveness of iohexol, and the data suggested that XP protected from CI-AKI without the diminish of the imaging effect of the contrast medium.

However, the present study does exist some limitations. (1) NIH suggests that it is better to include both male and female rats in the study to offset the gender differences, but in our study, we chose adult male rats only cause following the methods of other published documents. This gender bias might have an unknown influence on the final results to some extent. (2) We did not research deeply into the pharmacokinetics of XP, thus the characteristics of drug absorption, distribution, metabolism, and excretion of this antioxidant are unclear yet. Therefore, further optimization strategy in the drug administration is still obscure, thus what we can do is referring to the literature and early experience to administrate XP orally for seven days before modeling. (3) Oxidative stress has a close relation with mitochondrial damage because mitochondria are organelles that produce oxygen and supply energy. However, further interaction between them has not been elucidated deeply in the present research.

In conclusion, our present study provided evidence that XP may protect against iohexol-induced kidney apoptosis. Further, its anti-apoptotic effect may be mainly attributed to ameliorating the damage of mitochondrial morphology and function, intracellular and mitochondrial antioxidant activities. Also, XP suppressed the generation of fibrosis in the longer term, and all the above protective mechanisms of XP were irrelevant to the reduction of contrast imaging effect. The results suggested that antioxidant XP intervention could be a promising therapeutic approach for CI-AKI.

## Data Availability

The original contributions presented in the study are included in the article/Supplementary Material, further inquiries can be directed to the corresponding authors.
