# Corrigendum: A Novel Antioxidant Protects Against Contrast Medium-Induced Acute Kidney Injury in Rats

**DOI:** 10.3389/fphar.2021.724416

**Published:** 2021-07-09

**Authors:** Shuo Huang, Yanyan Tang, Tianjun Liu, Ning Zhang, Xueyan Yang, Dingwei Yang, Ge Hong

**Affiliations:** ^1^ Clinical College of Orthopedics, Tianjin Medical University, Tianjin, China; ^2^ Tianjin Key Laboratory of Biomedical Materials, Institute of Biomedical Engineering, Chinese Academy of Medical Science and Peking Union Medical College, Tianjin, China; ^3^ School of Chemical Engineering, Anhui University of Science and Technology, Huainan, China; ^4^ Department of Nephrology, Tianjin Hospital, Tianjin, China

**Keywords:** CI-AKI, apoptosis, oxidative stress, mitochondrial dysfunction, antioxidant

In the published article, there was an error in **Affiliation 1**. Instead of “^1^Clinical College of Orthopedics, Tianjin Medical University, Tianjin Hospital, Tianjin, China,” it should be “^1^Clinical College of Orthopedics, Tianjin Medical University, Tianjin, China.”

The authors apologize for this error and state that this does not change the scientific conclusions of the article in any way. The original article has been updated.

