# Supplementary figures and images for: A Novel Antioxidant Protects Against Contrast Medium-Induced Acute Kidney Injury in Rats
Source: Front Pharmacol. 2020 Nov 27;11:599577. doi: 10.3389/fphar.2020.599577 (PMC7729082; doi:10.3389/fphar.2020.599577)

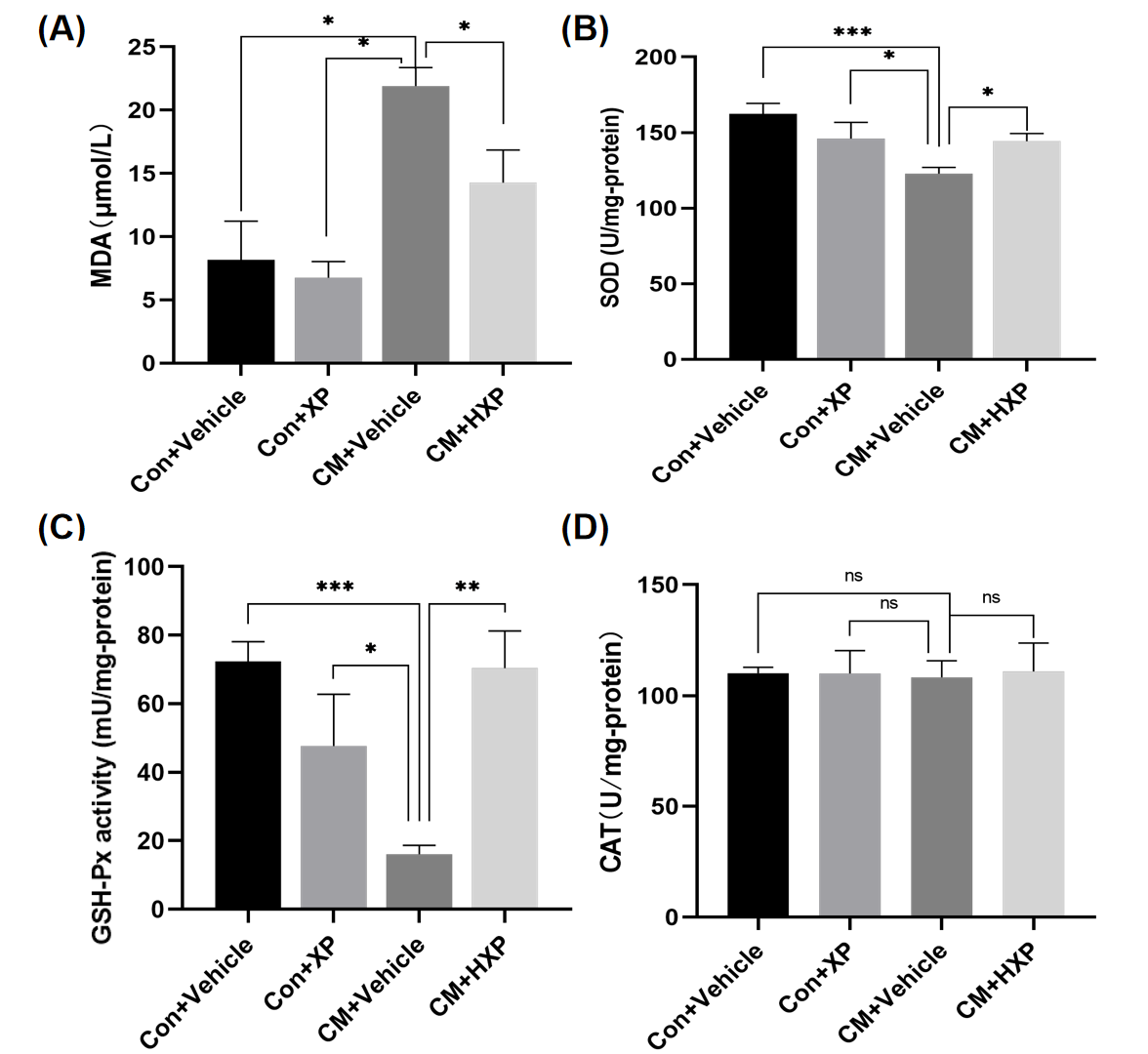

Supplement: Supplementary file 1 [file image2.tif]

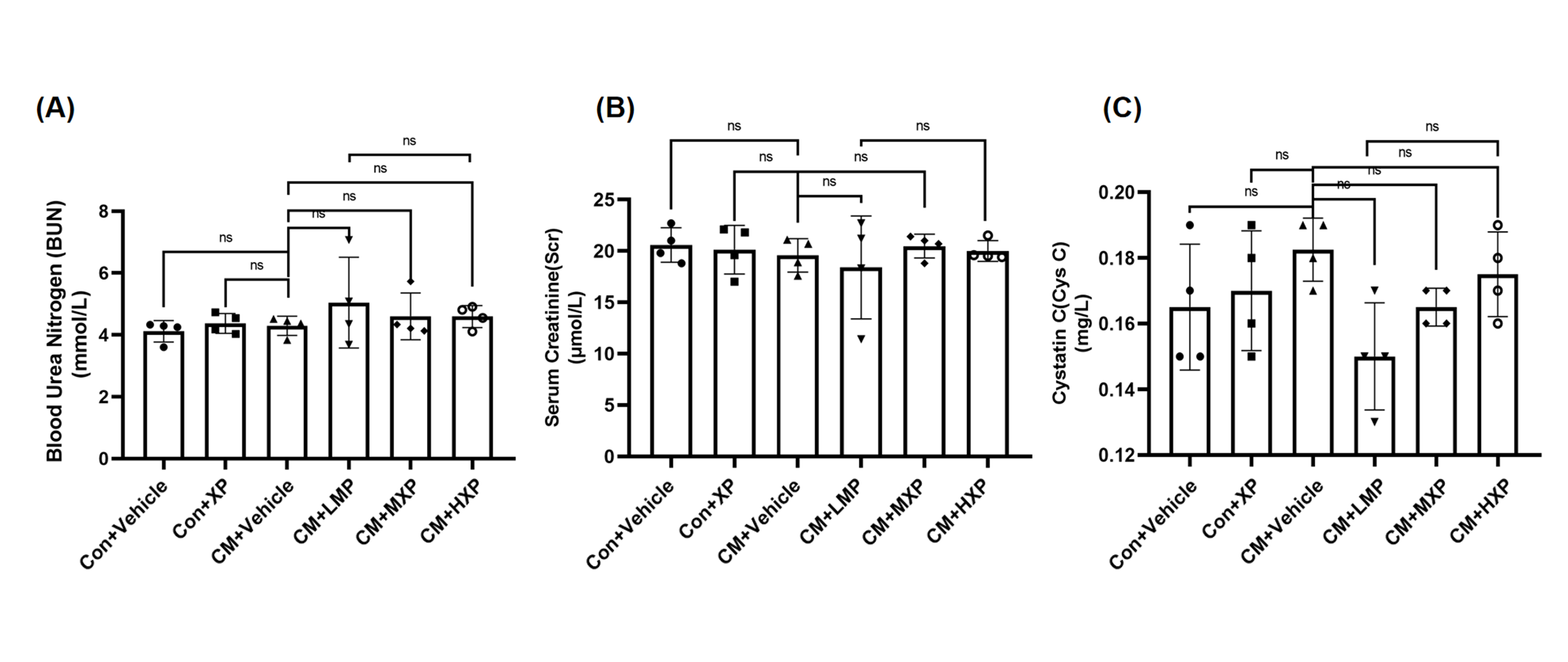

Supplement: Supplementary file 2 [file image1.tif]
